# Distinguishing Intraventricular Hemorrhage From Infectious Etiologies in Febrile Late Preterm Neonates: Case Studies in Antibiotic Stewardship

**DOI:** 10.7759/cureus.96379

**Published:** 2025-11-08

**Authors:** Ashley Kapinos, Sabrina Rhoads, Panitan Yossuck, Vignesh Gunasekaran

**Affiliations:** 1 Pediatrics, West Virginia School of Osteopathic Medicine, Lewisburg, USA; 2 Neonatology, West Virginia University School of Medicine, Martinsburg, USA

**Keywords:** antimicrobial stewardship, bloody csf, fever, intraventricular hemorrhage, late preterm neonate

## Abstract

Intraventricular hemorrhage (IVH) is a well-recognized complication in preterm infants, but its presentation as fever can be easily overlooked, leading to extensive sepsis workups and delayed diagnosis. This case report presents two late preterm infants who developed fever as the primary presenting symptom of IVH. Distinguishing IVH-related fever from that due to infectious causes can be challenging in this vulnerable population, where overuse of antimicrobials carries significant risks, including altered microbiome development, necrotizing enterocolitis, and antimicrobial resistance. Incorporating early diagnostic evaluation for IVH through cranial ultrasound in late preterm infants presenting with unexplained fever may avoid unnecessary tests and promote judicious antimicrobial use while improving overall neonatal outcomes.

## Introduction

In neonatal intensive care units (NICUs), fever in late preterm infants typically initiates a standardized sepsis evaluation given the high morbidity associated with infectious etiologies in this population. This reflects a clinical paradigm in which infection is presumed until proven otherwise [1]. This protocol minimizes the risk of missed sepsis but creates a diagnostic dilemma when fever stems from noninfectious causes. The automatic pursuit of infectious workups in these settings can lead to unnecessary investigations, prolonged antimicrobial exposure, and delayed recognition of alternative diagnoses. Once perinatal risk factors have resolved, the pretest probability of true infection in this population is often modest, yet noninfectious etiologies such as central thermoregulatory disturbances, medication reactions, or hemorrhagic events are less frequently prioritized early in the diagnostic process.

Intraventricular hemorrhage (IVH) is a significant complication affecting premature infants, with an incidence of 13-34% in different centers and decreasing with advancing gestational age [2]. IVH most commonly presents with neurological manifestations such as apnea, blood pressure and heart rate instability, decreased muscle tone, or seizures, whereas fever as a presenting symptom is rarely recognized [3,4]. To evaluate the rarity of this presentation, a focused literature search of PubMed and Scopus was conducted for English-language reports published between 1990 and 2024 using the terms “intraventricular hemorrhage,” “fever,” “preterm infant,” and “neonate.” Very few reports identified fever as the initial manifestation of IVH, underscoring the novelty and clinical relevance of this observation [5-7].

The objective of this report is to describe two cases of late preterm neonates who presented with fever as the primary manifestation of IVH and to raise awareness that IVH may underlie fever in preterm infants, emphasizing the importance of early consideration.

## Case presentation

Case 1

**Figure 1:**
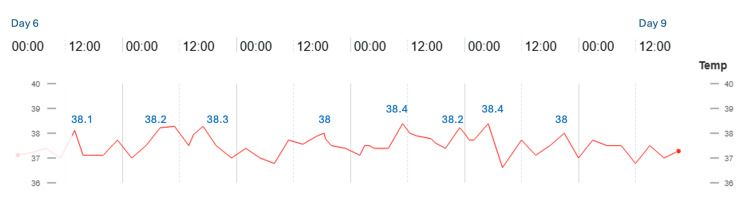
Temperature trend over time (Case 1) Data displayed in degrees Celsius

A 34-week female infant was born to a 25-year-old gravida 1, para 0 mother with multiple comorbidities, including preeclampsia, chronic hypertension, asthma, major depressive disorder, migraines, obesity, and factor V Leiden thrombophilia. The infant was delivered vaginally following induction because of maternal pre-eclampsia. The infant was admitted to the NICU on nasal continuous positive airway pressure (CPAP) of 5 and fraction of inspired oxygen (FiO_2_) of 0.25 for respiratory support and later transitioned to high-flow nasal canula (HFNC) and weaned to room air. On day 6 of life, the infant developed fever (Figure 1), measured via rectal thermometer, prompting a full sepsis evaluation.

The fever persisted for three days and was treated with acetaminophen. While blood culture remained negative and urinalysis was unremarkable, lumbar puncture revealed grossly bloody cerebrospinal fluid (CSF) with a white blood cell to red blood cell ratio of 1:475. CSF culture was negative. Additional testing for herpes simplex virus (HSV) was also negative. Respiratory viral panel testing was negative. Antibiotics and acyclovir were discontinued since the cultures and HSV testing were negative.

**Figure 2:**
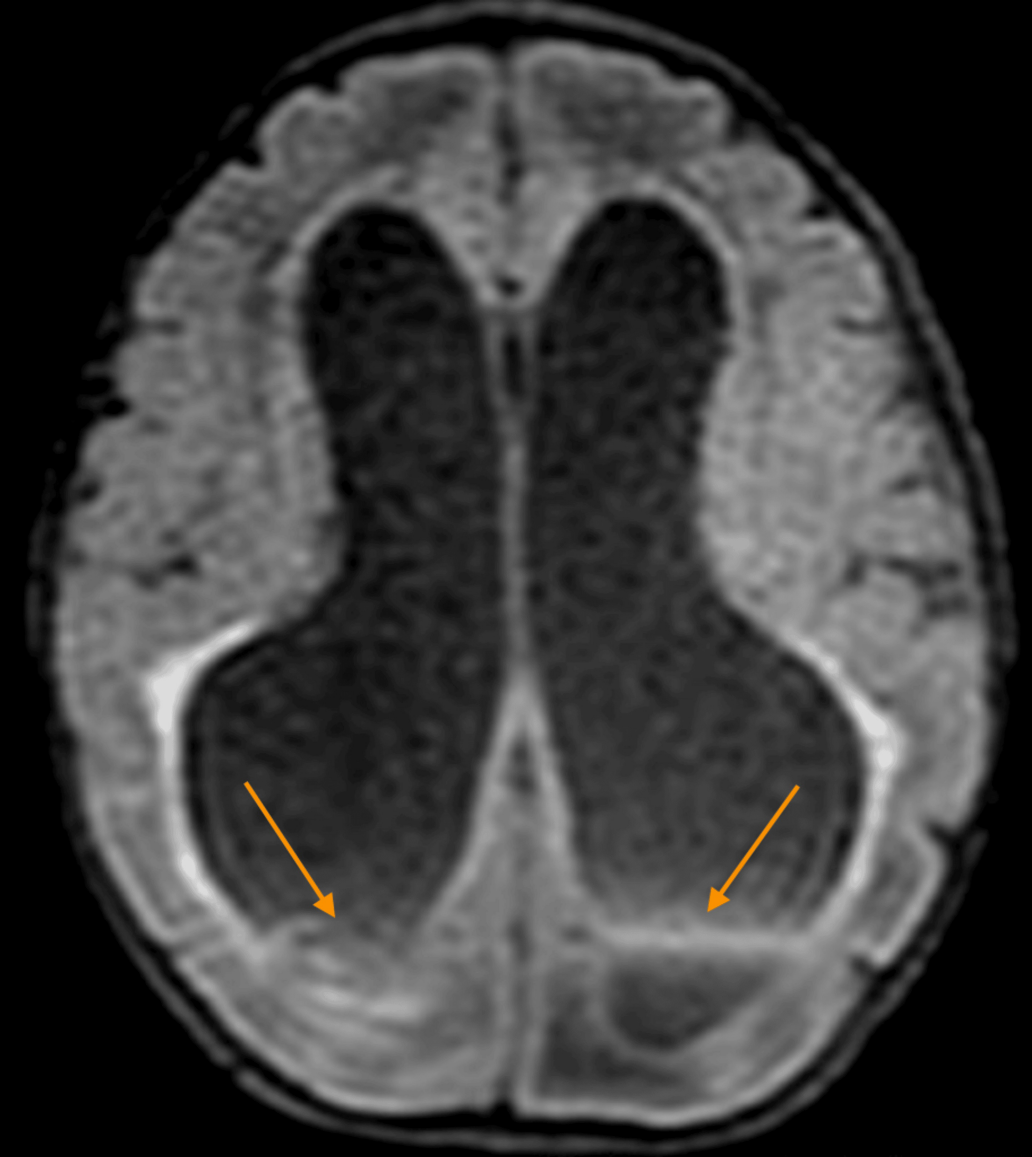
Axial T2 FLAIR MRI demonstrating a grade 3 intraventricular hemorrhage with associated ventriculomegaly (Case 1). Arrows show blood within the occipital horns of both ventricles, consistent with extensive bleeding and impaired cerebrospinal fluid drainage. FLAIR: fluid-attenuated inversion recovery

Given the fever and bloody CSF, head ultrasound was done, which revealed grade III IVH with associated ventriculomegaly. Serial head ultrasounds demonstrated progression to severe ventriculomegaly with increasing head circumference. Serial head circumference (HC) measurements showed an increase from 31.5 cm (1st percentile, day of birth) to 34 cm (9th percentile, day of life 18). MRI confirmed acute IVH with obstructive hydrocephalus (Figure 2). Due to worsening hydrocephalus, the infant was transferred to a tertiary center where a ventriculoperitoneal shunt was placed. Post surgery, HC was 33 cm (1st percentile). The infant was discharged home and continued to have appropriate developmental milestones at follow-up at four months of life. Patient labs and the clinical course are summarized in Table 1. 

**Table 1:** Patient labs and the clinical course for Case 1 CPAP: continuous positive airway pressure; CSF: cerebrospinal fluid; IVH: intraventricular hemorrhage; VP: ventriculoperitoneal

Day of Life	Clinical Presentation	Investigation/Findings	Intervention/Outcome
0	Late preterm (34 weeks), respiratory distress, CPAP started	Chest X-ray: Mild haziness; Stable vital signs	NICU admission
2	Increased oxygen needs, apnea	Labs unremarkable	Caffeine initiated, maintained on CPAP
6	Fever, temperature instability, initiated sepsis workup	Lumbar puncture: Bloody CSF; WBC:RBC ratio in CSF is 1:475 [Normal range is < 1:500]. Head US: Grade III IVH with ventriculomegaly [4]	Antibiotics, acyclovir started; MRI confirmed IVH.
9	Worsening hydrocephalus	Serial head circumference increase	Antibiotics and acyclovir discontinued. Transferred to tertiary center for neurosurgery evaluation
14	Progressive ventriculomegaly	MRI/angiography/venogram: obstructive hydrocephalus	VP shunt placed
Discharge/Follow-Up	Stable, meeting milestones	Routine pediatric and neurosurgery clinic visits	Continued follow up

Case 2

A 36-week male infant was born to a 21-year-old gravida 1, para 0 mother with a history of bacteriuria, marijuana use, gestational hypertension, and glucose intolerance. The mother presented with preeclampsia and underwent induction of labor. The infant was delivered via spontaneous vaginal delivery but experienced desaturation requiring CPAP support and NICU admission.

Initial presentation included mild haziness on chest X-ray consistent with respiratory distress syndrome, and a mild base deficit of -5 on arterial blood gas. A limited sepsis workup with blood culture was performed due to clinical illness and prolonged rupture of the membrane duration of about 18 hours. Empiric antibiotic therapy with ampicillin and gentamicin was discontinued after 48 hours following negative cultures.

On day 5 of life, the infant exhibited irritability and temperature elevation of 38.4°C, measured via rectal thermometer. Complete blood count (CBC) and C-reactive protein (CRP) were unremarkable. On day 6 of life, temperature again exceeded 38°C, prompting full sepsis evaluation, including lumbar puncture. Lumbar puncture revealed grossly bloody CSF with protein count of 231 mg/dl, RBC count of 173,350/µL, and nucleated cell count of 1,010/µL. The infant also had an elevated bilirubin of 15.3 mg/dL. The meningitis/encephalitis panel by polymerase chain reaction (PCR) analysis, and HSV testing in CSF and swabs were negative. Blood, urine, and CSF cultures reported no growth.

**Figure 3:**
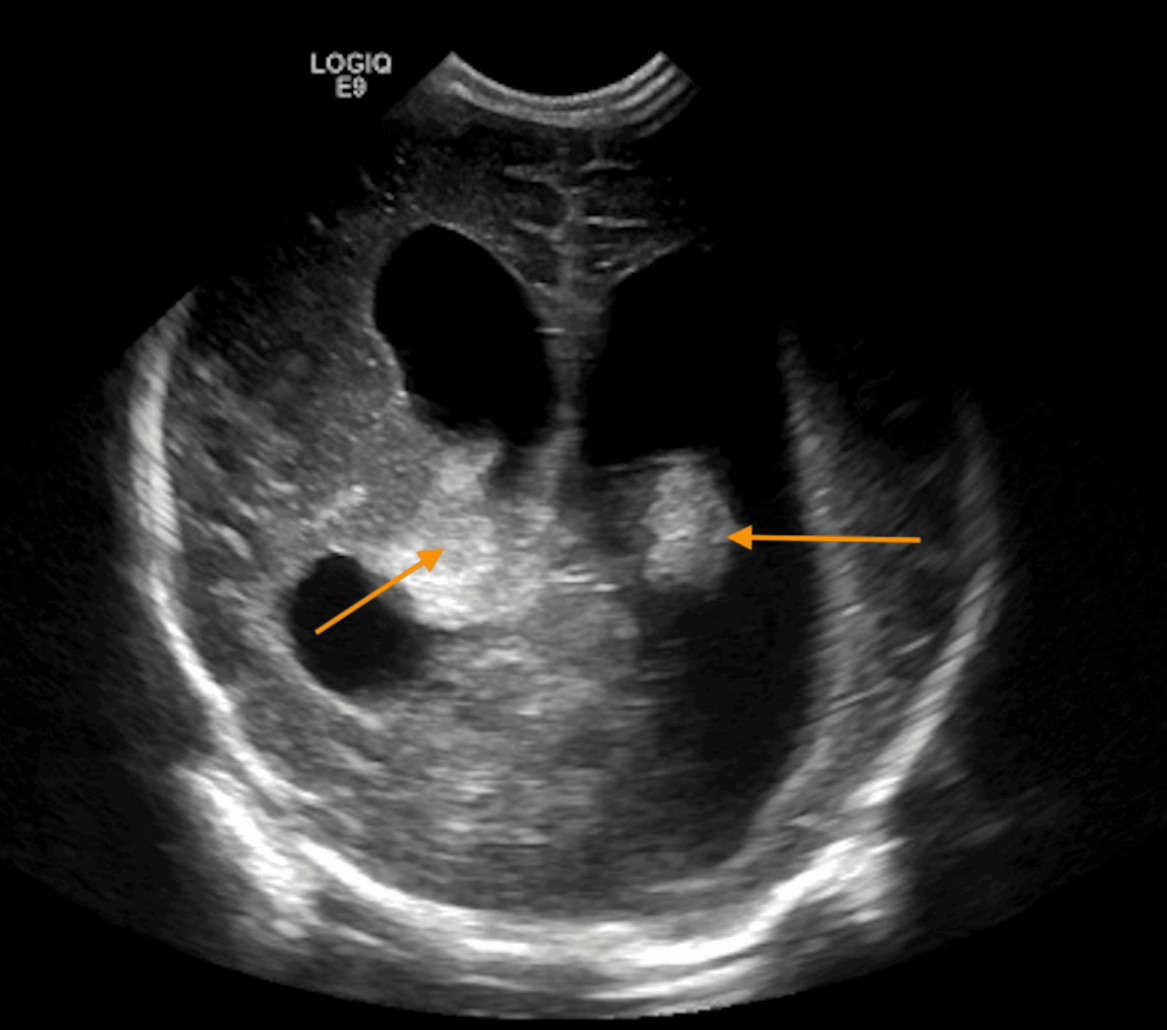
Coronal cranial ultrasound image (Case 2) demonstrating a grade 3 intraventricular hemorrhage and ventriculomegaly. Arrows indicate echogenic material within the dilated lateral ventricles, consistent with intraventricular blood.

Head ultrasound on day 7 revealed suspected bilateral grade II-III germinal matrix hemorrhages with developing hydrocephalus and ventriculomegaly (Figure 3). MRI confirmed IVH and ruled out abscess, parenchymal hemorrhage, or ischemia. The fever was treated with acetaminophen at 10 mg/kg/dose, and it resolved after four days. Empiric antimicrobial therapy and acyclovir were discontinued, given the absence of an infectious etiology. Serial HC done, which showed an increase in HC from 32.4 cm (5th percentile, day of birth ) to 35.5 cm (39th percentile, day of life 14), and then it remained stable. The infant remained clinically stable with no worsening hydrocephalus. Repeat MRI at three months and six months showed no worsening of hydrocephalus, and they continued reaching appropriate developmental milestones at follow-up per their pediatrician. Patient labs and the clinical course are summarized in Table 2. 

**Table 2:** Patient labs and the clinical course (Case 2) NICU; neonatal intensive care unit; CBC: complete blood count; CRP: C-reactive protein; IVH: intraventricular hemorrhage; BR: bilirubin; MRA/MRV: magnetic resonance angiography/magnetic resonance venography

Day of Life	Clinical Presentation	Investigation/Findings	Intervention/Outcome
0	Late preterm (36 weeks), respiratory distress, CPAP started	Chest X-ray: Mild haziness; Stable vital signs	NICU admission
2	Clinical improvement	Weaned to nasal cannula	Empiric antibiotics
4	Transitioned to room air	Cultures negative	Antibiotics discontinued
5	Fever, irritability, tremors	CBC/CRP: Unremarkable; Temp >38°C [Normal: 36.5 C – 37.5 C]	Sepsis workup initiated
6	Persistent fever	BR level of 15.3 mg/dL (Normal<16 mg/dl). LP: Bloody CSF: RBC count of 173,350/µL [Normal: <100/µL], protein count of 231 mg/dl [Normal: <150 mg/dl], and nucleated cell count of 1,010/µL [Normal: 15/µL – 30 /µL] Head ultrasound: Grade II-III IVH and ventriculomegaly [4]	Broad-spectrum antimicrobials started
7	Stable with fever	MRI: IVH confirmed, no abscess	Cultures negative; MRA/MRV ruled out thrombosis.
10	Stable, no worsening hydrocephalus	Antimicrobials discontinued	Routine follow-up
Discharge/Follow-Up	Stable, meeting milestones	Repeat MRI at 3 months: Stable ventricles	Routine pediatric and neurosurgery follow-up

## Discussion

These cases illustrate the importance of recognizing IVH as a potential cause of fever in late-preterm infants. The Papile grading system is used to classify the severity of IVH in both cases [8]. While sepsis remains the most common cause of fever in neonates, central fever due to intracranial hemorrhage should be considered in the differential diagnosis. Risk factors for IVH include prematurity, maternal pre-eclampsia, and perinatal complications. Studies have demonstrated that antenatal infection and maternal complications increase the risk of IVH in preterm infants [9].

Fever as a presenting symptom of IVH

Fever in the neonatal period is classically attributed to infectious causes, with its presence prompting a full sepsis workup and empirical antibiotic administration. However, IVH, though uncommon in late preterm and term infants, should remain a consideration in the differential diagnosis of neonatal fever. 

It is difficult to determine the true incidence of IVH in term neonates as they are often asymptomatic. Heibel et al. conducted cranial ultrasounds on 1,000 clinically normal term newborns and found that 2% exhibited signs of IVH [10]. When symptomatic, IVH can mimic that of sepsis: irritability, stupor, apnea, seizures [11]. Fever has also been documented as a presenting symptom. In 1973, Pomerance and Richardson reported a case series of three neonates whose first clinical sign of IVH was hyperpyrexia [5]. They also referenced 18 similar cases in which fever preceded any other symptoms of hemorrhage. 

In the current report, both late preterm infants were admitted to the NICU and presented with fever. This initial presentation led to a strong diagnostic bias toward infection, and the neonates were started on prophylactic antibiotic therapy. After extensive sepsis evaluations, there was no evidence of infection. However, neuroimaging ultimately revealed IVH with hydrocephalus in both cases. Pediatric infectious disease consultants initially recommended a full 14-day course of empirical antibiotics due to persistent fever and diagnostic uncertainty. Pediatric neurology was also consulted. At the time, neither subspecialty was familiar with IVH as a potential cause of fever in late preterm neonates. Following a review of available literature and multidisciplinary discussion, antibiotics were discontinued after confirming negative cultures. These cases highlight a critical gap in provider awareness across specialties and the need for broader education on the atypical presentation of IVH in this population.

This misattribution of fever to infection rather than hemorrhage not only delays accurate diagnosis but also exposes neonates to prolonged antibiotic use and unnecessary interventions. Fang et al. reported a significantly higher incidence of intracranial hemorrhage among febrile neonates compared to afebrile ones, with 39.3% of intracranial hemorrhage (ICH) cases presenting with hyperthermia [6]. Moreover, in most of these cases, no infectious etiology was identified, suggesting that ICH itself may well be the source of fever. 

Mechanism of fever in IVH 

The exact pathophysiology by which IVH leads to fever remains complex and multifactorial. One proposed mechanism involves the breakdown of red blood cells within the ventricles, leading to the release of hemoglobin and subsequent conversion of arachidonic acid to prostaglandins, particularly PGE2 and PGF2α, which have been shown to be key mediators of fever [12]. Experimental models show that injection of hemoglobin into the cerebrospinal fluid raises core body temperature via increased CSF PGE2 levels. Additionally, inflammatory cytokines such as interleukin-6 and tumor necrosis factor alpha (TNF-α), released in response to blood products in the CSF, further contribute to the febrile response. Disruption of hypothalamic thermoregulatory centers due to hemorrhage, particularly in deep brain structures, may also lead to dysregulated body temperature control [13]. 

Importance of early neuroimaging 

Current neonatal protocols often exclude routine cranial ultrasonography in late preterm or term infants unless there are specific clinical concerns. In our patients, if the diagnostic evaluation had halted at sepsis workup, the diagnosis of IVH would have been missed. Both cases underscore the diagnostic value of early neuroimaging in febrile neonates, even in the absence of overt neurologic symptoms. As Fang et al emphasized, IVH often lacks significant external manifestations and may go undetected without imaging [6]. 

Impact of early diagnosis on management and neurodevelopment

Timely identification of IVH has direct implications for clinical management and long-term outcomes. Early diagnosis allows for the cessation of unnecessary antibiotic therapy, monitoring for complications such as hydrocephalus, and planning for appropriate neurodevelopmental follow-up. A study of outcomes with infants with known IVH demonstrated the importance of early identification of it, citing one death and three infants left severely handicapped after delayed diagnosis [14]. In one recently published case, a neonate with isolated fever was later found to have bilateral grade 3 IVH and ultimately required a ventriculoperitoneal shunt for progressive hydrocephalus [7]. Serial head circumference measurements and follow-up imaging are critical components of post-diagnosis care to mitigate neurodevelopmental sequelae and to set the neonate up for long-term success. 

Literature review of existing reports 

Although IVH is a recognized complication in preterm infants, its presentation as isolated fever without overt neurological signs is exceedingly rare. Our literature review did not identify any previous reports of such a presentation in late preterm neonates, making this case series a novel contribution to neonatal neurology and infectious disease differential diagnosis. One retrospective study of 153 febrile term neonates identified ICH in 11 cases (7.2%), none with infectious causes, and found that 39.3% of all ICH cases had fever as the initial symptom [6]. Another report described the case of a term infant with isolated fever, later diagnosed with bilateral IVH, reinforcing the diagnostic challenge and rarity of this presentation [7]. A case review by Pomerance and Richardson suggests that fever can be the sole presenting symptom [5]. These cases align with our own and add weight to the argument for heightened clinical suspicion and earlier imaging in similar scenarios. 

## Conclusions

This case series highlights the critical importance of considering IVH as a potential cause of fever in late preterm infants, particularly when CSF is bloody and cultures remain negative. These cases support the addition of early cranial ultrasound consideration in late-preterm neonates with unexplained fever to accelerate diagnostic clarification and guide antimicrobial decisions. Early recognition of IVH in such scenarios may reduce unnecessary and prolonged antibiotic exposure, supporting current principles of antimicrobial stewardship.

Implementing timely cranial imaging could potentially guide appropriate diagnosis and intervention, which may ultimately improve neonatal outcomes. Clinicians should maintain a high index of suspicion for non-infectious etiologies of fever to optimize care and limit unwarranted antibiotic use in this vulnerable population. Future research is warranted to determine whether early neuroimaging measurably reduces antibiotic exposure and improves outcomes in late preterm infants presenting with fever of noninfectious origin.
